# A Ratiometric Fluorescence Detection Method for Berberine Using Triplex-Containing DNA-Templated Silver Nanoclusters

**DOI:** 10.3390/molecules29153459

**Published:** 2024-07-24

**Authors:** Ming Zhu, Mingyang Sun, Juntong Liu, Changbao Chen, Yonggang Yang, Ye Teng

**Affiliations:** School of Pharmacy, Changchun University of Chinese Medicine, 1035 Boshuo Road, Changchun 130117, China

**Keywords:** berberine, DNA-AgNCs, triplex, ratiometric fluorescence

## Abstract

Berberine (BBR), as a natural isoquinoline alkaloid, has demonstrated various pharmacological activities, and is widely applied in the treatment of diseases. The quantitative analysis of BBR is important for pharmacological studies and clinical applications. In this work, utilizing the specific interaction between BBR and triplex DNA, a sensitive and selective fluorescent detecting method was established with DNA-templated silver nanoclusters (DNA-AgNCs). After binding with the triplex structure in the template of DNA-AgNCs, BBR quenched the fluorescence of DNA-AgNCs and formed BBR-triplex complex with yellow–green fluorescence. The ratiometric fluorescence signal showed a linear relationship with BBR concentration in a range from 10 nM to 1000 nM, with a detection limit of 10 nM. Our method exhibited excellent sensitivity and selectivity, and was further applied in BBR detection in real samples.

## 1. Introduction

BBR is a natural isoquinoline alkaloid, which is considered the major effective content of many Chinese medical herbs, such as *Coptis chinensis* and *Phellodendron amurense* [1]. It has been widely used in pharmacological studies and disease treatment due to its multiple pharmacological activities, including anti-inflammatory, antimicrobial, and anticancer activities, and so on [2]. For example, new synthesis methods and structural modifications of BBR and its derivatives have been attempted to improve the bioavailability, as well as to elucidate the anti-inflammatory pathway and structure—activity relationships [3,4]. Researchers also found that the mechanism of BBR in lipid-lowering and insulin resistance was related to its impact on the gastrointestinal microbiota [5]. Moreover, BBR was reported to significantly inhibit the migration and invasion of cancer cells by down-regulating the expression of specific genes or pathways [6]. Interestingly, BBR was demonstrated to have good binding affinity with specific structures of DNA/RNA, such as duplex [7,8], triplex [8,9], G-quadruplex [10], and i-motif [11], which expanded its applications in biological fields. For example, BBR was considered as a promising G-quadruplex stabilizer which suppressed cancer and viral gene expression [10]. Xu et al. developed label-free DNA-based logic gates utilizing the specific interaction between BBR and i-motif [11].

The multiple functions and clinical effects made quantitative analysis of BBR important for its mechanism study. Various analytical methods for BBR quantification have been established, including colorimetric assay [12], chemiluminescence [13], fluorescence [14,15], electrochemical analysis [16], high-performance liquid chromatography (HPLC) [17], mass spectrometry [18], resonance light scattering technique (RLS) [19], etc. However, many of these detection methods suffered from complicated sample pretreatments, complex operations, expensive instruments, time-consuming processes, and poor sensitivity or selectivity. Notably, detecting methods with excellent fluorescent probes are favorable in BBR quantification because of stable fluorescence signal, simple sample preparation, fast detection speed, high sensitivity, and low cost. Well-designed novel fluorescent probes are desired.

Metal nanoclusters, such as silver nanoclusters (AgNCs) and gold nanoclusters (AuNCs), are a class of fluorescent probes with nanoscale and strong fluorescence, which have been widely applied in the detection of biomolecules and cell imaging [20,21,22,23]. Among them, AgNCs are characterized by their excellent photophysical properties, low cost, good biocompatibility, and low toxicity [24]. Especially, DNA-AgNCs have attracted researchers interest because of their excellent fluorescence properties and the design capability of DNA templates. The excitation/emission wavelengths of DNA-AgNCs could be altered by changing the sequences of DNA templates [25]. In addition, the specific bindings between DNA sequences and biomolecules could greatly improve the selectivity of detecting methods based on DNA-AgNCs. Therefore, various DNA-AgNC probes have been designed and applied in the detection of DNAs/RNAs, metal ions, enzymes, proteins, and other active biological molecules [26,27].

Herein, a novel fluorescent probe was designed for BBR detection utilizing the intercalative binding of BBR and DNA triplex structure. According to previous reports [8], BBR partially intercalated in triplex, specifically stabilized the Hoogsteen base pairing in the third strand of triplex. In this work, the designed probe consisted of a hairpin structure with a loop containing six cytidines (C6-loop). The C6-loop was used for fluorescent DNA-AgNC synthesis. The stem in the hairpin structure formed triplex in the presence of a single complementary strand, providing intercalative binding sites for BBR. The formation of the BBR–triplex complex significantly quenched the fluorescence of DNA-AgNCs and an increasing emission of BBR–triplex complex was found. The ratiometric fluorescence signal was correlated to BBR concentration in a range from 10 nM to 1000 nM. A sensitive and selective detecting method was achieved with a detection limit of 10 nM. It was further applied in the detection of BBR in real samples and obtained accurate and reliable results.

## 2. Results

### 2.1. Characterization of Triplex-Containing DNA-AgNCs

**Figure 1:**
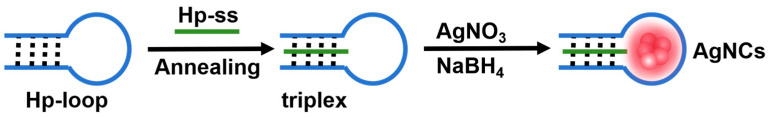
The schematic demonstration of the synthesis of triplex-containing DNA-AgNCs.

**Figure 2:**
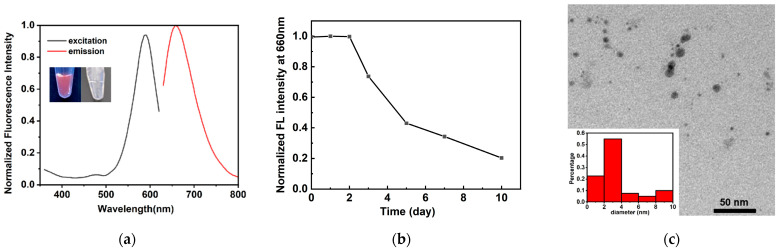
(**a**) The excitation and emission spectra of DNA-AgNCs (inset: the photos of DNA-AgNCs under UV light (254 nm) and sun light respectively); (**b**) the normalized fluorescence intensity at 660 nm after synthesis for 0, 1, 2, 3, 5, 7 and 10 days; (**c**) the TEM image of DNA-AgNCs (inset: size distribution of DNA-AgNCs).

A triplex-containing template was designed for DNA-AgNC synthesis, which was composed of a hairpin-structured DNA (Hp-loop1, Table 1) and a short complementary sequence (Hp-ss1, Table 1). Figure 1 shows a schematic diagram of the DNA template structure and the synthesis of DNA-AgNCs. Hp-loop1 contained a C6-loop for the protection of AgNCs and a full-matched stem for triplex formation with Hp-ss1. The DNA-AgNCs were synthesized in sodium phosphate buffer (PB) at pH 6.0, according to previous reports [28]. Briefly, AgNO_3_ and NaBH_4_ were added to DNA solution in a final ratio of 1:6:6. The obtained DNA-AgNCs were transparent and colorless, and exhibited red emission under UV light (excitation wavelength: 254 nm), as shown in the inset photos of Figure 2a. Figure 2a shows that the fluorescence excitation and emission spectra of synthesized DNA-AgNCs. A red fluorescence emission at 660 nm was detected when DNA-AgNCs were excited at 590 nm. The fluorescence of DNA-AgNCs remained stable for two days, and gradually decreased to about 20% after 10 days (Figure 2b). The sizes of DNA-AgNCs were characterized by TEM (Figure 2c). Most of synthesized DNA-AgNCs were smaller than 10 nm, and the average diameter of DNA-AgNCs was 3.60 nm.

**Figure 3:**
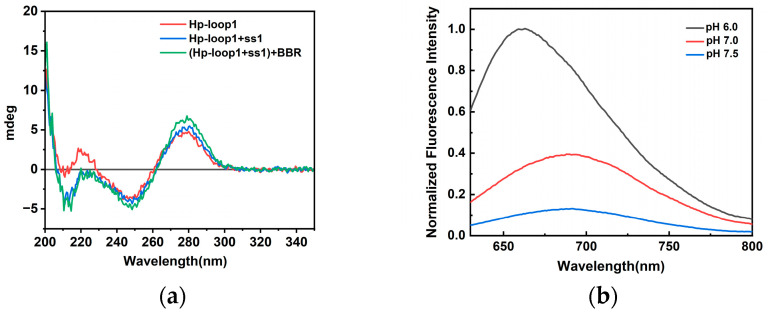
(**a**) The CD spectra of Hp-loop1, Hp-loop1 + ss1 and Hp-loop1 + ss1 + BBR; (**b**) the fluorescence emission spectra of (Hp-loop1 + ss1)-AgNCs in the buffers at pH 6.0, 7.0 and 7.5.

The structure of template was characterized by CD spectra, as shown in Figure 3a. The Hp-loop1 showed a negative peak at 255 nm, and a positive peak at 275 nm, demonstrating the formation of a hairpin structure. In the presence of Hp-ss1, a negative peak at 215 nm existed, suggested triplex formation in the stem region. Considering the effect of pH on triplex formation, the fluorescence of DNA-AgNCs were compared in PB buffers at pH 6.0, 7.0, and 7.5, respectively (Figure 3b). DNA-AgNCs synthesized in pH 6.0 had better fluorescence intensity, which may be due to the fact that AgNCs preferred to locate in C6-loop rather than triplex formed in the stem under acidic condition.

### 2.2. The Interaction between DNA-AgNCs and BBR

**Figure 4:**
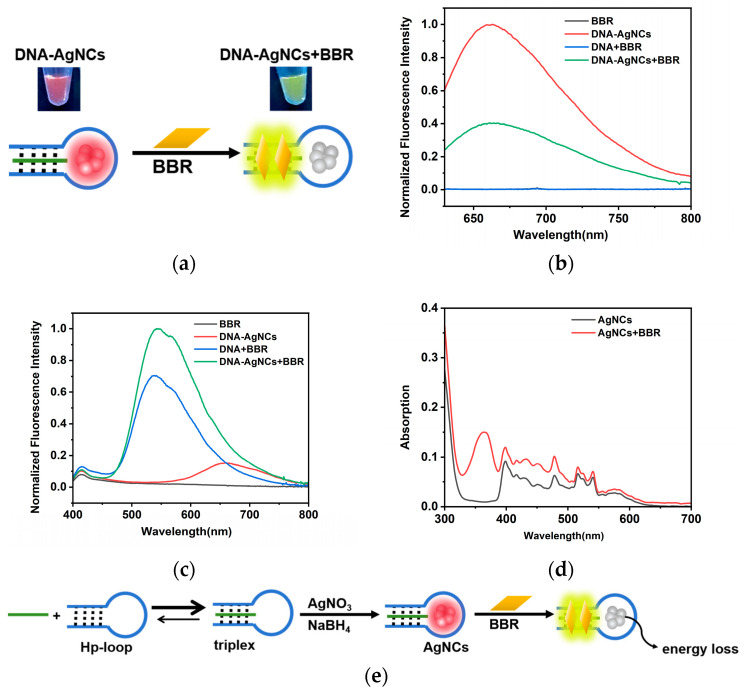
(**a**) The schematic demonstration of the interaction between BBR and DNA-AgNCs and the photos of DNA-AgNCs in the absence (left) and presence (right) of BBR under UV light (excitation wavelength: 245 nm); (**b**) the fluorescence emission spectra of BBR, DNA-AgNCs, DNA+BBR and DNA-AgNCs+BBR excited by 590 nm; (**c**) the fluorescence emission spectra of BBR, DNA-AgNCs, DNA+BBR and DNA-AgNCs+BBR excited by 360 nm; (**d**) the UV–Vis absorption of DNA-AgNCs in the absence (black) and presence (red) of BBR; (**e**) the schematic demonstration of the equilibrium between duplex and triplex and the possible mechanism of the ratiometric fluorescence changes.

To verify the potential application of synthesized DNA-AgNC probes in BBR detection, the interactions between DNA-AgNCs and BBR were investigated. As shown in Figure 4a, after the addition of BBR, an obvious color change from red to yellow green was observed under UV light (excitation wavelength: 245 nm). The fluorescence of DNA-AgNCs at 660 nm were quenched (Figure 4b), and an emission at 560 nm excited by 360 nm was greatly enhanced (Figure 4c). The fluorescence quenching at 660 nm was considered to be related to the structural changes in the stem region. According to previous reports [25,28], the fluorescence of DNA-AgNCs were highly dependent on their surrounding environment, especially the structure of DNA. The combination of BBR and triplex in the stem region stabilized triplex structure, which might cause the fluorescence quenching at 660 nm. No fluorescence at 560 nm was detected in the absence of BBR or triplex, suggesting that this emission peak was generated from the combination of BBR and triplex, which was consist with previous reports [29]. It could also be demonstrated by the fluorescence at 560 nm in the presence of 5 μM BBR and different concentrations of DNA-AgNCs. As shown in Figure S1, the fluorescence enhanced with the increase in DNA-AgNC concentration. When the concentration was higher than 500 nM, the derivative of the curve decreased, indicating a saturation of DNA–BBR interaction at high concentration. In addition, DNA-AgNCs + BBR showed a higher fluorescence intensity at 560 nm compared to DNA + BBR, as shown in Figure 4c, indicating that more BBRs were combined with DNA in the presence of DNA-AgNCs. There was a reversible equilibrium between duplex and triplex structures, as the tension of C-loop and the partially protonated cytosines in weak acidic environment. During the synthesis of DNA-AgNCs, the binding of C-Ag^+^-C reduced the loop tension, resulting in the equilibrium shifting towards the formation of triplex. More triplexes provided more binding sites for BBR, resulting the stronger fluorescence at 560 nm comparing to DNA alone.

The UV–Vis spectra of DNA-AgNCs in the absence and presence of BBR correspond to the fluorescence results (Figure 4d). DNA-AgNCs had an absorption peak at 590 nm, which was consistent with their fluorescence excitation wavelength. After the addition of BBR, a new absorption peak appeared at 360 nm, corresponding to the fluorescence spectra of the BBR–triplex complex. In addition, the absorption peak of DNA-AgNCs had no obvious change, suggested that no aggregation of DNA-AgNCs happened. This was consistent with our speculation that the fluorescence quenching mechanism was related to the environmental change around DNA-AgNCs, and the interaction between BBR and triplex might cause the absorbed energy lost in a nonluminescence form. Figure 4e is an illustration of the possible mechanism of the ratiometric fluorescence probe based on our experiments.

**Figure 5:**
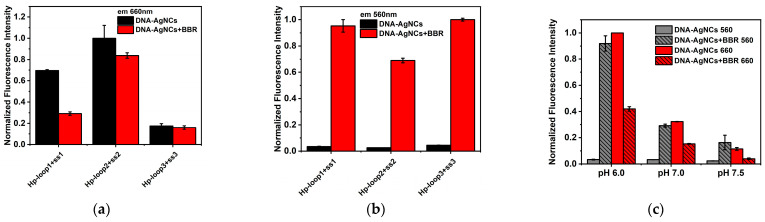
(**a**) The normalized fluorescence intensity at 660 nm of (Hp-loop1 + Hp-ss1)-AgNCs, (Hp-loop2 + Hp-ss2)-AgNCs and (Hp-loop3 + Hp-ss3)-AgNCs in the absence and presence of BBR at pH 6.0; (**b**) the normalized fluorescence intensity at 560 nm of (Hp-loop1 + Hp-ss1)-AgNCs, (Hp-loop2 + Hp-ss2)-AgNCs and (Hp-loop3 + Hp-ss3)-AgNCs in the absence and presence of BBR at pH 6.0; (**c**) the normalized fluorescence intensity of (Hp-loop1 + Hp-ss1)-AgNCs at 560 nm and 660 nm in the absence and presence of BBR in PB buffers at pH 6.0, 7.0 and 7.5.

Considering the interaction between BBR and triplex was highly dependent on the content of T•A-T (•: Hoogsteen base pairing; -: Watson–Crick base pairing) [8,9], two similar triplex-containing templates with same length but different T•A-T contents (Hp-loop2 + Hp-ss2 and Hp-loop3 + Hp-ss3 in Table 1) were designed and utilized for AgNCs synthesis. Comparing with Hp-loop1+Hp-ss1, Hp-loop2 + Hp-ss2 had two more T•A-T triplets, while Hp-loop3 + Hp-ss3 had two fewer T•A-T triplets. The fluorescence of synthesized DNA-AgNCs and the effect of BBR on DNA-AgNCs fluorescence were shown in Figure 5a,b. Compared to (Hp-loop1 + Hp-ss1)-AgNCs, (Hp-loop2 + Hp-ss2)-AgNCs exhibited equivalent fluorescence intensity, but less impact on their fluorescence after the addition of BBR. (Hp-loop3 + Hp-ss3)-AgNCs had the weakest fluorescence among them. A possible explanation was that cytosine had better affinity to silver ions, and the high content of cytosine in the stem region of Hp-loop3 + Hp-ss3 led to less silver ions binding in C6-loop region and resulted in weak fluorescence of synthesized AgNCs. On the other hand, the fluorescence intensity at 560 nm was mainly dependent on the content of T•A-T (Figure 5b) because of the intercalation of BBR in T•A-T triplets. Overall, the ratiometric fluorescence signal was mainly determined by the fluorescence property of DNA-AgNCs among three different templates, and (Hp-loop1 + Hp-ss1)-AgNCs were the better probes in BBR detection as a consequence.

In addition, the best pH condition for BBR interaction was also optimized. The synthesis of DNA-AgNCs and the interaction with BBR were carried out in the buffers at pH 6.0, 7.0, and 7.5, respectively. The results were shown in Figure 5c. Consistent with previous studies, an acidic environment was more favorable for the formation of the triplex, leading to better fluorescence of both DNA-AgNCs and the BBR–triplex complex.

### 2.3. Detection of BBR

**Figure 6:**
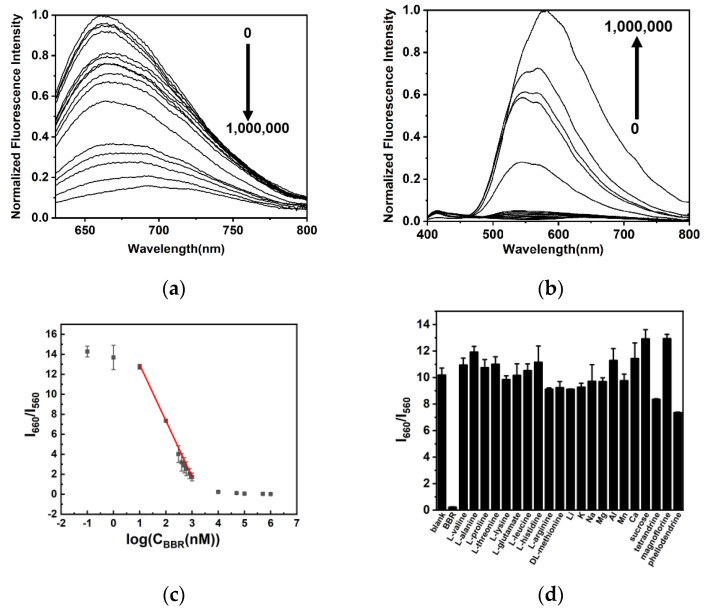
(**a**) The fluorescence spectra of DNA-AgNCs excited by 590 nm in the presence of different concentrations of BBR; (**b**) the fluorescence spectra of DNA-AgNCs excited by 360 nm in the presence of different concentrations of BBR; (**c**) the relationship between ratiometric fluorescence intensity I_660_/I_560_ and BBR concentration; (**d**) the ratiometric fluorescence intensity I_660_/I_560_ in the presence of BBR, different amino acids, cations, sucrose, tetrandrine, magnoflorine and phellodendrine at a same concentration.

Under the optimal condition, DNA-AgNCs were mixed with different concentrations of BBR and incubated for 30 min at room temperature, and the fluorescence intensity at 560 nm and 660 nm were recorded. As shown in Figure 6a,b, with the increasing concentration of BBR, the fluorescence at 560 nm enhanced and the fluorescence at 660 nm quenched. The ratiometric fluorescence intensity I_660_/I_560_ exhibited an excellent linear relationship (I_660_/I_560_= −5.60 log(C_BBR_) + 18.6, R^2^ = 0.997) to the logarithmic values of BBR concentration (Figure 6c). The linear range was from 10 nM to 1000 nM, and the detection limit was 10 nM.

The selectivity of our sensing method was further investigated. Several common coexisting substances and ions in biological samples were tested for comparison, including L-valine, L-alanine, L-proline, L-threonine, L-histidine, L-glutamic, L-leucine, L-lysine, L–arginine, DL-methionine, Li^+^, K^+^, Na^+^, Mg^2+^, Zn^2+^, Al^3+^, Mn^2+^, Ca^2+^ and sucrose. Especially considering the structural similarity of alkaloids and the potential application of our method in drug identification, three other alkaloids including tetrandrine, magnoflorine and phellodendrine were also detected for comparison. As shown in Figure 6d, all of these substances present limited effects on I_660_/I_560_, suggesting that our sensing method present an excellent selectivity on BBR.

Our sensing method was further applied in the detection of BBR in real samples. BBR tablets were prepared into solutions with a certain concentration, and the concentration of BBR was detected by both DNA-AgNC probes and HPLC. The results were listed in Table 2. The average content of BBR per tablet measured by DNA-AgNCs was 29.80 mg, while the value measured by HPLC was 29.23 mg. These results were close, and both of them were consistent with the labeled amount of 30 (±4.5) mg. This demonstrated that our detecting method was able to apply to BBR detection in real samples with a good accuracy.

## 3. Discussion

DNA-AgNCs are promising fluorescence probes due to their excellent fluorescence property, low cost, easy synthesis, good biocompatibility, and low toxicity. Compared to traditional detection methods, our method does not require complex sample processing or large amounts of organic solvents. The fluorescence detection has a short analysis time, and has the potential to finish a large numbers of samples in a short time, improving the analysis efficiency. Especially, as a powerful tool, template DNA can be well-designed to achieve better detection results. Normal fluorescence quenching methods often suffer from the interference from the quenching effect of coexisting substances, resulting in false positive results. The ratiometric fluorescence method can greatly improve this problem, enhancing the sensitivity and selectivity and make them resistant to environmental interference. In this work, utilizing the interaction between triplex structure and BBR, triplex-containing DNA-AgNCs were designed to construct a novel detecting method for BBR. As a result, a sensitive and selective ratiometric fluorescence method was obtained. The detection limit was 10 nM, which is lower than or equivalent to most fluorescent BBR detection methods.

In addition, the DNA template of our probe has good biocompatibility, and is easy to synthesize and modify. Our triplex-containing probe has a short single strand for triplex formation, which can be further modified with different aptamers to improve the targeting ability to specific targets or cells. It has great potential in cell imaging and in vivo detection. Our probes have been applied in the detection of real samples, and they are expected to apply in pharmacological studies as in vivo probes in the future, providing new ideas for mechanism studies and new drug development.

## 4. Materials and Methods

### 4.1. Oligodeoxynucleotides and Materials

All DNA sequences in this work were purchased from Shanghai Sangon Biotechnology Co., Ltd. (Shanghai, China), and used without further purification. The sequences are listed in Table 1. DNA samples were dissolved in distilled water purified by Thermo Type 2 system (Langenselbold, Germany), and stored in −20 °C before use. Silver nitrate and sodium borohydride were purchased from Sigma-Aldrich (Darmstadt, Germany). Other reagents were obtained from Shanghai Yuanye Biotechnology Co., Ltd. (Shanghai, China). All solution were prepared in distilled water.

### 4.2. Synthesis of DNA-AgNCs

In total, 10 μM of template DNA in sodium phosphate buffer (PB, 0.2 M, pH = 6.0) was incubated at 90 °C for 5 min and then cooled from 90 °C to room temperature. The DNA sample mixed with 60 μM of AgNO_3_ and incubated at room temperature for 10 min. A total of 60 μM of fresh NaBH_4_ was later added with vigorous shaking for 10 min. The solution was kept in the dark at 4 °C for 24 h before fluorescence characterization.

### 4.3. Characterization of DNA-AgNCs

The UV–Visible spectra of DNA-AgNCs were recorded by Aurora-900 ultramicro spectrophotometer (Hangzhou Haipei Instrument Co., Ltd., Zhejiang, China). The sizes of DNA-AgNCs were characterized by a H-600 transmission electron microscope (Hitachi, Ltd., Tokyo, Japan). Circular dichroism (CD) spectra were collected by a JASCO J-820 spectropolarimeter (JASCO Corporation, Tokyo, Japan).

### 4.4. Fluorescence Measurement

Fluorescence of DNA-AgNCs were performed with a SpectraMax Paradigm Multi-Mode Detection Platform system (Molecular Devices, San Jose, CA, USA) with a final concentration of 1 μM. As the fluorescence intensity of synthesized DNA-AgNCs varied within a reasonable range, the maximum intensity of each synthesized DNA-AgNCs was normalized to 1 for the convenience of understanding. For BBR detection, the prepared DNA-AgNCs solutions were mixed with different concentrations of BBR, and the fluorescence was recorded after 0.5 h incubation.

### 4.5. Real Sample Detection

The DNA-AgNCs were applied in the detection of BBR in Compound Berberine Tablets (BBR tablets, Hubei Nordsheng Pharmaceutical Co., Ltd., Xiangyang, China). Fifteen BBR tablets were crushed and ground, weighed, and divided into three parts of powder on average. The three parts of powder were, respectively, dissolved and filtered into a solution of proper concentration. The accurate concentration of BBR solution was further determined by fluorescence with DNA-AgNCs and HPLC (Agilent 1260), respectively. For HPLC detection, the chromatographic column was a ZORBAX SB-C18 column (4.6 mm × 250 mm, 5 μm). The separation was performed by isocratic elution at a flow rate of 1.0 mL/min at 30 °C, using a mobile phase of 25:75 acetonitrile and KH_2_PO_4_ (0.01 M). The injection volume was 10 μL, and the detection wavelength was 265 nm.

## 5. Conclusions

In conclusion, a simple, sensitive and selective fluorescence detecting method of BBR was constructed with triplex-containing DNA-AgNCs. Utilizing the specific interaction between triplex structure and BBR, the red fluorescence of DNA-AgNCs was quenched and the complex of triplex-BBR emitted yellow green fluorescence. The ratiometric fluorescence intensity exhibited a linear relationship with BBR concentration in a range from 10 to 1000 nM, with a limit of detection of 10 nM. This method has been used in real sample detection, and it is promising for in vivo analysis due to the easy modification and strong specificity of DNA.

## Figures and Tables

**Table 1:** DNA sequences used in this work.

Name	Sequences(5′-3′)
Hp-loop1	CTTTCTTCCTTCCCCCCAAGGAAGAAAG
Hp-ss1	TTCCTTCTTTC
Hp-loop2	CTTCCTTCCCTCCCCCCAGGGAAGGAAG
Hp-ss2	TCCCTTCCTTC
Hp-loop3	CTTTTTTCTTTCCCCCCAAAGAAAAAAG
Hp-ss3	TTTCTTTTTTC

**Table 2:** The comparison of BBR contents in BBR tablets detected by our ratiometric fluorescent probes and HPLC.

Method	BBR Content (mg)	Average Content (mg)	RSD (%)
Ratiometric fluorescence with DNA-AgNCs	29.90	29.80	2.71
	30.64		
	29.02		
HPLC	29.23	29.23	0.07
	29.26		
	29.25		

## Data Availability

The original contributions presented in the study are included in the article/Supplementary Materials, further inquiries can be directed to the corresponding authors.

## Supplementary Materials

The following supporting information can be downloaded at: https://www.mdpi.com/article/10.3390/molecules29153459/s1, Figure S1: The relationship of normalized fluorescence intensity at 560 nm (black) and its derivative (red) with the logarithm of DNA-AgNCs concentration.
