# Supplementary material for: A Ratiometric Fluorescence Detection Method for Berberine Using Triplex-Containing DNA-Templated Silver Nanoclusters
Source: Molecules. 2024 Jul 24;29(15):3459. doi: 10.3390/molecules29153459 (PMC11314519; doi:10.3390/molecules29153459)
Supplement: Supplementary file 1 [file molecules-29-03459-s001.zip › molecules-3081869-supplementary.pdf]

Supporting Information

A ratiometric fluorescence detection method for berberine using triplex-containing DNA-templated silver nanoclusters

Ming Zhu, Mingyang Sun, Juntong Liu, Changbao Chen, Yonggang Yang,* and Ye Teng,*

School of Pharmacy, Changchun University of Chinese Medicine,

1035 Boshuo Road, Changchun 130117, China

Email: tengye@ccucm.edu.cn

Figure

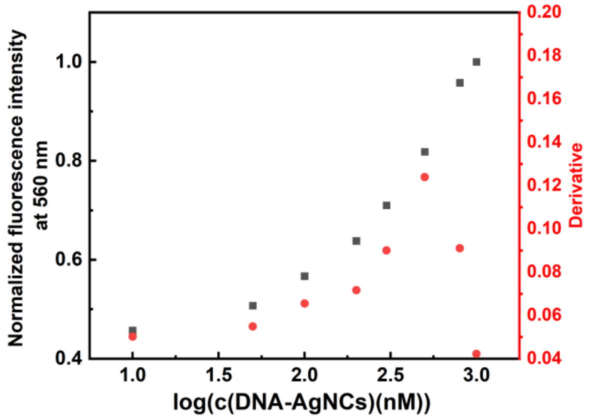

Figure S1. The relationship of normalized fluorescence intensity at 560 nm (black) and its derivative (red) with the logarithm of DNA-AgNCs concentration.
